# Antimicrobial Resistance in Gram-negative bacteria from Urinary Specimens: a study of prevalence, risk factors and molecular mechanisms of resistance (ARGUS) in Zimbabwe – a study protocol

**DOI:** 10.12688/wellcomeopenres.15977.1

**Published:** 2020-06-12

**Authors:** Ioana D. Olaru, Shunmay Yeung, Rashida A. Ferrand, Richard Stabler, Prosper Chonzi, David Mabey, Heidi Hopkins, John Bradley, Kudzai P.E. Masunda, Shungu Munyati, Katharina Kranzer

**Affiliations:** 1 Clinical Research Department, London School of Hygiene & Tropical Medicine, London, WC1E 7HT, UK; 2 Biomedical Research and Training Institute, Harare, Zimbabwe; 3 Department of Health, Harare City Council, Harare, Zimbabwe

**Keywords:** AMR, antibiotic resistance, Escherichia coli

## Abstract

Antimicrobial resistance (AMR) is compromising our ability to successfully treat infections. There are few data on gram-negative AMR prevalence in sub-Saharan Africa especially from the outpatient setting. This study aims to investigate the prevalence of and underlying molecular mechanisms for AMR in gram-negative bacilli causing urinary tract infections (UTIs) in Zimbabwe. Risk factors for AMR and how AMR impacts on clinical outcomes will also be investigated.

Adults presenting with UTI symptoms at primary health clinics in Harare will be included. A questionnaire will be administered, and urine samples will be collected for culture. Participants with positive urine cultures will be followed up at 7-14 days post-enrolment. All participants will also be followed by telephone at 28 days to determine clinical outcomes.

Bacterial identification and antibiotic susceptibility testing will be performed on positive cultures.

The results from this study will be used to inform policy and development of treatment recommendations. Whole genome sequencing results will provide a better understanding of the prevalent resistance genes in Zimbabwe, of the spread of successful clones, and potentially will contribute to developing strategies to tackle AMR.

## Introduction

Antimicrobials have revolutionized modern medicine leading to important reductions in mortality, morbidity and disability. Their discovery and use in medical practice was, however, accompanied by the rapid development of resistance ^1^. Antimicrobial resistance (AMR) can reverse the benefits brought by these drugs, leading to increased patient deaths and healthcare costs ^2, 3^. Considering the current trends of increasing AMR, it is estimated that by 2050, 10 million deaths per year globally will be caused by antimicrobial resistant infections, exceeding the number of deaths due to cancer ^4^.

The increase in AMR is mainly driven by inappropriate antimicrobial use in humans and animals and insufficient infection control systems. Exposure to antimicrobials selects for spontaneous mutations or the acquisition and propagation of bacterial clones harbouring resistance genes ^5^. Resistance genes are then mobilized and can disseminate to other commensal and pathogenic organisms ^6^. This in turn may lead to increased carriage of resistant organisms in the population and an increase in use of second-line antimicrobial drugs ^7^. At an individual level, other risk factors for infections due to resistant organisms are underlying co-morbid conditions and healthcare contact ^8^.

**Figure 1.**
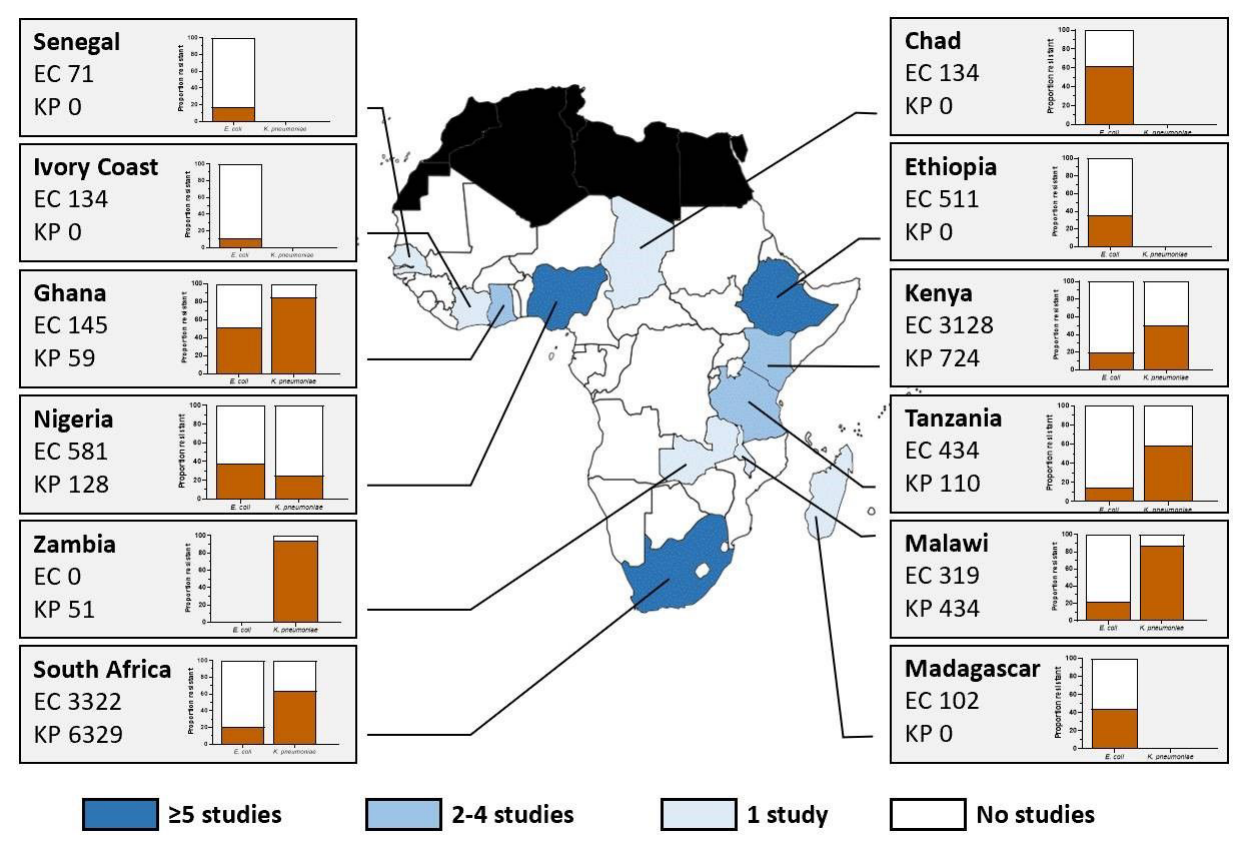
Studies from sub-Saharan Africa and prevalence of third-generation cephalosporin resistance in *E. coli* and *K. pneumoniae.* Only studies describing antimicrobial resistance prevalence in isolates from blood or urine cultures are included. The numbers in the small boxes represent the number of isolates with a reported third-generation cephalosporin test result. The orange bars in the graphs represent the proportion of isolates with third-generation cephalosporin resistance for E. coli (left) and K. pneumoniae (right). The white bars represent the proportion of susceptible isolates. The shaded boxes at the bottom of the picture represent the number of studies according to country. EC: Escherichia coli; KP: Klebsiella pneumoniae. One study presented data from multiple countries and was not included on the map. This figure was compiled using the studies listed in Table 1.

AMR is a global problem affecting all countries irrespective of income and geographical location ^9^. However, countries differ widely with regards to their detection and reporting capabilities. Surveillance plays a key role in understanding the epidemiology of AMR and informs interventions and control measures. Global surveillance networks, such as the Global AMR Surveillance System (GLASS), were established to ensure standardised data collection and analysis and facilitate data sharing regionally and globally. However, thus far few African countries contribute data to these networks ^10^, and the WHO Africa region has limited AMR prevalence data ^9, 11, 12^. GLASS focuses on a number of priority pathogens including *Escherichia coli* and *Klebsiella pneumoniae* isolated from priority specimens such as blood and urine ^13^. Figure 1 and Table 1 illustrate the lack of data from sub-Saharan Africa on AMR in key pathogens, as well as the high prevalence of resistance where such data are available.

**Table 1.** Prevalence of resistance in *E. coli* and *Klebsiella spp.* in Sub-Saharan Africa in studies published since 2013. AST antimicrobial susceptibility testing; BSAC British Society for Antimicrobial Chemotherapy; CA community acquired; CLSI Clinical and Laboratory Standards Institute; EUCAST European Committee on Antimicrobial Susceptibility Testing; HA hospital acquired HCA healthcare associated; ISO International Organization for Standardization; LIMS laboratory information management system; QC quality control; UTI urinary tract infection.

Author (year)	Country	Study period	Setting	Patient population	Laboratory type	AST method	Sample type	Number of isolates tested	Quality assurance
Apondi *et al.* [2016] ^15^	Kenya	2002–2013, retrospective	One teaching/ referral public hospital	Inpatients, all blood culture isolates with *K. pneumoniae* most from neonatal unit	Hospital laboratory	CLSI, automated blood culture, disc diffusion	blood	281 *K.* *pneumoniae*	ISO accredited laboratory, internal QMS
Ayoyi *et al.* [2017] ^16^	Kenya	NA, prospective	Antenatal clinics from informal settlements in Nairobi	Booking appointment for antenatal clinics, asymptomatic	Not specified	CLSI, disc diffusion	urine	85 *E. coli*	Not specified
Barry *et al.* [2017] ^17^	Senegal	08/2012- 03/2013, prospective	Urban hospitals and primary clinics	Outpatients and inpatients admitted <72h; CA-infections	Not clearly specified	Automated (Vitek) for identification and AST	urine	74 *E. coli*	Not specified
Derbie *et al.* [2017] ^18^	Ethiopia	01/2012- 12/2014, retrospective	Referral hospital, health centers, private clinics	Not clearly specified, no antibiotics <2 weeks	Research laboratory, referral center	CLSI, Biochemical tests for identification, disc diffusion	urine	72 *E. coli*	Not specified
Eibach *et al.* [2016] ^19^	Ghana	09/2007- 07/2009; 01/2010- 12/2012, prospective	Regional hospital (rural)	Inpatients of all ages presenting with fever, neonates with suspected sepsis; CA-infections and HAI in neonates	Not specified	EUCAST; Automated blood culture, biochemical identification confirmed by MALDI-TOF abroad; AST by Vitek2	Blood	50 *E. coli*	Not specified
Kaduma *et al.* [2019] ^20^	Tanzania	03/2017- 05/2017, prospective	Regional referral hospital	Pregnant women with and without pre-eclampsia attending antenatal clinics or admitted in a matched case- control design; no symptoms of UTI	Not specified	CLSI, AST by disc diffusion	urine	50 *E. coli*	Use of reference strains for culture and AST
Malande *et al.* [2019] ^21^	South Africa	01/2005- 12/2014, retrospective	Major urban tertiary-level referral hospital for children	Children admitted to hospital; 47% CA- infections; 28% HAI; 24% HCA	External hospital laboratory	CLSI, automated blood culture, identification and AST using Vitek2	blood	583 *E. coli*	Not specified
Manyahi *et al.* [2017] ^22^	Tanzania	06/2004- 01/2005, retrospective	Tertiary care hospital	Inpatients and outpatients presenting to the hospital; 49% HAI; 51% CA	Hospital laboratory	CLSI; identification using biochemical tests and Vitek2	urine	110 *E. coli*	Not specified
Nyasulu *et al.* [2017] ^23^	South Africa	07/2005- 12/2009, retrospective	7 tertiary public hospitals with laboratories linked to the National Health Laboratory Service	Samples from hospitals	Hospital laboratories reporting to a central laboratory	CLSI, automated blood cultures, identification with Vitek2, or Microscan or conventional biochemical methods, AST manual (disc diffusion or Etest) or automatic (Vitek2 or microscan)	blood	4466 *K.* *pneumoniae*	The individual hospital laboratories had good QA practices, and computarised LIMS
Obeng- Nkrumah *et al.* [2016] ^24^	Ghana	01/2010- 12/2013, retrospective	Tertiary-care teaching hospital	Inpatients who had a blood culture collected and were aged >28 days; either presenting to the hospital directly or referred for investigations	Hospital laboratory	CLSI, automated blood cultures, identification using biochemical tests, AST by disc diffusion	blood	118 *E. coli* 63 *K.* *pneumoniae*	Not specified
Oli *et al.* [2017] ^25^	Nigeria	05-07/2016, prospective	Regional teaching hospital	Women with symptoms of UTI presenting to various outpatient clinics	Laboratory within university	CLSI, biochemical tests for identification, AST by disc diffusion	urine	61 *E. coli*	Not specified
Perovic *et al.* [2018] ^26^	South Africa	01-12/2016, retrospective	Not specified	Not specified	Four private laboratories	CLSI	blood	2781 *E. coli* 2466 *K.* *pneumoniae*	Not clearly specified, accredited laboratories
Seni *et al.* [2019] ^27^	Tanzania	07/2016- 10/2017, prospective	4 referral hospitals urban and rural	Children presenting to the hospitals with suspected blood stream infections	Hospital laboratory and university laboratory	CLSI; manual blood cultures, AST by disc diffusion	blood	55 *K.* *pneumoniae*	Use of reference strains
Seni *et al.* [2019] ^28^	Tanzania	03/2016- 05/2017, prospective	7 healthcare facilities: tertiary hospital, regional referral hospital, district hospital, health centers	Pregnant women, inpatients and outpatients	Not specified	CLSI, biochemical identification, AST by disc diffusion	urine	164 *E. coli* 55 *K.* *pneumoniae*	Use of reference strains for culture, biochemical tests and AST
Toy *et al.* [2019] ^29^	Burkina Faso, Ethiopia, Ghana, Guinea-Bissau, Kenya, Madagascar, Senegal, Sudan, Tanzania	01/2010- 09/2013, prospective	12 healthcare facilities from the 9 participating countries	Patients of all ages presenting with fever as in- or outpatients; CA-infections	Laboratories at each site	CLSI; Mostly automated blood cultures, one setting with manual; biochemical identification;AST by disc diffusion	blood	120 *E. coli* 89 *Klebsiella* *spp.*	IQA established for the study, use of reference strains; central coordinator performing QA and working towards establishing a QMS; isolates sent to reference laboratories for confirmation
Tuem *et al.* [2019] ^30^	Ethiopia	01/2012- 12/2017 retrospective	Tertiary hospital	Patients attending the microbiology laboratory	Laboratory of the hospital	NCCLS; AST by disc diffusion	urine	148 *E. coli* 50 *K.* *pneumoniae*	Not specified
Elikwu *et al.* [2017] ^31^	Nigeria	12/2015- 04/2016, prospective	University teaching hospital	In- and outpatients with suspected UTIs, all ages	Laboratory of the hospital	CLSI, AST by disc diffusion	urine	96 E. coli	Not specified
Onoh *et al.* [2013] ^32^	Nigeria	04/2010- 03/2011, prospective	2 tertiary referral hospitals, district-level – antenatal clinic	Pregnant women with UTI symptoms presenting to the antenatal clinics, outpatients	Not specified	Not specified, manual (disc diffusion)	urine	128 *E. coli*	Not specified
Oladeinde *et al.* (2015) ^33^	Nigeria	06/2011- 11/2011, prospective	Antenatal clinics at traditional birth center	Pregnant women, asymptomatic presenting for antenatal care, outpatients	Not specified	BSAC, not specified	urine	51 *E. coli*	Not specified
Omoregie *et al.* (2013) ^34^	Nigeria	02/2010- 01/2011, prospective	University, referral hospital	Neonatal sepsis	Not specified	BSAC, manual (disc diffusion)	blood	74 *K.* *pneumoniae*	Not specified
Iregbu *et al.* (2013) ^35^	Nigeria	01/2010- 12/2012, prospective	Tertiary hospital	Not clearly specified, suspected UTI, mostly outpatients; likely most were CA- infections	Microbiology laboratory of the national hospital	Not specified, manual (disc diffusion)	urine	323 *E. coli* 202 *K.* *pneumoniae*	Not specified
Abejew *et al.* (2014) ^36^	Ethiopia	09/2002- 09/2011, retrospective	Public and private hospitals, primary care centers	Not clearly specified, inpatients and outpatients	Regional health research laboratory	National standards, manual (disc diffusion)	urine	410 *E. coli*	Use of reference strains for AST
Akingbade *et al.* (2014) ^37^	Nigeria	Not specified, unclear	Inpatient and outpatient clinics from public health facilities	Inpatients and outpatients	Not specified	National standards, manual (disc diffusion)	urine	120 *E. coli*	Use of reference strains for AST
Moroh, *et al.* (2014) ^37^	Ivory Coast	2000–2011, retrospective	Not clearly specified	Inpatients and outpatients	Central laboratory of a teaching hospital	Not specified, manual (disc diffusion)	urine	879 *E. coli* (345 from inpatients, 534 from outpatients)	Use of reference strains
Eshetie *et al.* (2015) ^38^	Ethiopia	02/2014- 05/2014, prospective	1 teaching referral hospital	Inpatients and outpatients with symptomatic UTI	Accredited referral laboratory	CLSI, manual (disc diffusion)	urine	112 *E. coli*	Clearly describes QC, use of reference strain for media
Olorunmola *et al.* (2013) ^39^	Nigeria	05/2003- 12/2005, unclear	2 tertiary care hospitals	Inpatients and outpatients with suspected UTI	Not specified	Not specified, manual (disc diffusion, agar dilution)	urine	137 *E. coli*	Not specified
Buys *et al* (2016) ^40^	South Africa	01/2006- 12/2011, retrospective	1 public teaching and referral hospital for children	Inpatients, children, CA 5%, HA 86% and HCA 9% infections	Laboratory in the national health laboratory service	CLSI, automated culture, identification and AST (Vitek), (disc diffusion and Etests)	blood	409 *K.* *pneumoniae*	Has a LIMS
Kabwe *et al.* (2016) ^41^	Zambia	10/2013- 05/2014, prospective	NICU of a teaching hospital	Inpatients, neonates with suspected sepsis	Not specified	CLSI, automated culture, manual ID and AST (disc diffusion)	blood	77 *K.* *pneumoniae*	Not specified
Maina *et al.* (2016) ^42^	Kenya	09/2010- 2014, retrospective	1 teaching hospital, internationally funded	Inpatients and outpatients attending a private hospital (middle- and high- income); mixed between CO and hospital-onset	Hospital laboratory	CLSI, mainly automated culture and identification and AST (Vitek), if required manual (disc diffusion, E-tests)	Blood and urine	2912 *E. coli* (urine) 139 *E. coli* (blood) 365 *K.* *pneumoniae* (urine) 83 *K.* *pneumoniae* (blood)	ISO accredited, data extracted from LIMS
Mamuye *et al.* (2016) ^43^	Ethiopia	08/2013- 01/2014, prospective	1 teaching tertiary hospital	Inpatients and outpatients	Not specified	CLSI, manual (disc diffusion)	urine	53 *E. coli*	Reference strains for AST
Bitew *et al.* (2017) ^44^	Ethiopia	05/2015- 05/2016, prospective	private laboratory	Suspected UTI	Private laboratory	Not specified, automated (Vitek)	urine	135 *E. coli*	Reference strains for AST
Henson *et al* (2017) ^45^	Kenya	Since 1994, retrospective	District hospital with international funding	Inpatients adults and children with CA (49%) and HA infections (51%)	In-hospital laboratory, international funding	CLSI, automated culture; semi- automated (broth microdilution with automated reading)	blood	198 *K.* *pneumoniae*	Not specified, samples collected within ongoing surveillance for invasive infections
Kengne et al (2017) ^46^	Chad	07-11/2014, prospective	1 general hospital	Inpatients and outpatients	Not specified	Not specified, automated (Vitek)	urine	128 *E. coli*	Reference strain
Lochan et al (2017) ^47^	South Africa	01/2011- 12/2012, retrospective	1 tertiary care paediatric hospital	Inpatients with CA (36%), HA (54%) and HCA infections (10%)	National health laboratory	CLSI, automated culture; automated identification and AST (Vitek)	blood	92 *E. coli* 92 *K.* *pneumoniae*	Not specified
Rakotovao- Ravahatra et al (2017) ^48^	Madagascar	01/2014- 10/2016, retrospective	1 teaching hospital	Not specified	Laboratory of the teaching hospital	French standards, manual (disc diffusion)	urine	102 *E. coli*	Not specified
Forson *et al.* (2018) ^49^	Ghana	02-08/2016, prospective	5 hospitals	Asymptomatic pregnant women	Not specified	CLSI, manual (disc diffusion)	urine	82 *E. coli*	Use of reference strains
Iroh Tam *et al.* (2019) ^50^	Malawi	1998–2017, retrospective	1 large central hospital with international funding	Inpatients, reported data on children under 5 years with suspected sepsis	Hospital laboratory with international funding	BSAC, manual and automated blood cultures, biochemical tests for identification, AST by disc diffusion	blood	1998–2017: 857 *E. coli* 578 *K.* *pneumoniae* *2008–2012:* 163 *E. coli* 132 *K.* *pneumoniae* *2013–2017:* 165 *E. coli* 316 *K.* *pneumoniae*	Not specified, part of an institutional blood culture surveillance

Due to limited availability of diagnostics, insufficient laboratory capacity and suboptimal funding of health care systems, in sub-Saharan Africa infections are often treated using a “syndromic” approach ^14^. Samples for microbiological investigations are rarely collected outside of national tuberculosis, malaria and HIV programmes and a few academic centers. Consequently, there is an overuse of antimicrobials resulting from a “just-in-case” approach to treating infections and a lack of resistance data on which to base prescribing ^51^. This is the case of ceftriaxone, which is commonly prescribed to patients admitted to hospitals with suspected infections ^52, 53^. The AWaRe classification is a framework developed by the WHO for categorizing essential antimicrobials as ‘watch’, ‘access’ or ‘reserve’ and for guiding their prescription and usage. According to this classification ceftriaxone, the most widely available third-generation cephalosporin, is in the “watch” group and should be used judiciously for restricted indications ^54^.

Third-generation cephalosporins are essential drugs for the treatment of severe bacterial infections. Resistance usually develops through the acquisition of extended-spectrum beta-lactamases (ESBL), which hydrolyze the beta-lactam ring rendering third-generation cephalosporins ineffective ^55^. ESBL genes are transferrable between different species of *Enterobacteriaceae* and also are often associated with other mechanisms that cause fluoroquinolone, aminoglycoside and sulphonamide resistance, thus leading to resistance to the main classes of antimicrobials used to treat Gram-negative infections ^56^. In sub-Saharan Africa, access to amikacin or to carbapenems for treatment of third-generation cephalosporin resistant infections is extremely limited and cost-prohibitive ^57^.

Over two thirds of the 37.9 million people living with HIV (PLWH) are in sub-Saharan Africa ^58^. Southern Africa is particularly severely affected with most countries having an adult HIV prevalence exceeding 10% ^58^. PLWH attend health care facilities frequently for scheduled and unscheduled visits, receive more antimicrobial prescriptions and experience more hospital admissions than people without HIV, and therefore may be at increased risk for infections with antimicrobial-resistant organisms ^59, 60^.

Co-trimoxazole prophylaxis has been shown to reduce mortality and hospital admissions in PLWH ^61^, and is currently recommended for all children and adults with advanced HIV or who are at risk for malaria and severe bacterial infections ^62^. However, co-trimoxazole has been reported to increase carriage of resistant organisms in PLWH ^63, 64^. The increase in colonization with resistant organisms is not limited to an increase in co-trimoxazole resistance but also extends to other drug classes including cephalosporins and fluoroquinolones ^65, 66^. For Gram-negative bacilli (GNB), this may be due to the co-localization of resistance genes on the same mobile genetic elements which are transferrable between bacterial species ^67^.

Urinary tract infections (UTIs) are the most common infections caused by GNB, with an estimated incidence of 10 per 100 person years among women ^68^. Resistance patterns of GNB causing UTIs reflect the community burden of resistance with the added advantage that samples are easy to obtain, and processing easy to standardise ^69^. *E. coli* is the most common cause of UTIs especially in the community setting and *K. pneumoniae* the second most common ^70^.

## Protocol

### Study hypotheses

This study hypothesizes that among patients presenting with symptoms of UTI, PLWH have a higher risk of infections with resistant organisms than individuals without HIV. Additionally, because of the AMR prevalence in this setting, the current first-line treatment recommendations of amoxicillin or norfloxacin for UTI treatment will be suboptimal in terms of bacterial antimicrobial susceptibility and resolution of infection.

### Study aims and objectives

The aims of this study are i) to determine if infections in PLWH are more commonly due to antimicrobial resistant organisms, compared with infections in individuals without HIV infection; ii) to explore the prevalence of and underlying molecular mechanisms for AMR in GNB causing UTIs, iii) to investigate risk factors for AMR, and iv) to examine how AMR impacts on clinical outcome.

*Primary objective:*

1 To determine if there is an association between HIV status and infections with ESBL-producing *E. coli* in adults (aged 18 years or older) who present with symptoms of UTI to primary healthcare services in Harare, Zimbabwe.

*Secondary objectives:*

2 To estimate the prevalence of third-generation cephalosporin resistance due to ESBL production in *E. coli* isolated from individuals who present with symptoms of UTI to primary healthcare services in Harare;
3 To determine the prevalence of resistance to amoxicillin and quinolones (first-line drugs for UTIs according to Zimbabwean National guidelines ^71^) in bacteria causing UTIs;
4 To identify the risk factors associated with UTIs with bacteria resistant to amoxicillin and quinolones, and with ESBL-producing and multidrug-resistant bacterial strains;
5 To determine the impact of resistance to first-line antimicrobials (amoxicillin and ciprofloxacin) on clinical outcomes (defined as complete resolution of symptoms at follow-up);
6 To evaluate the causes of negative urine cultures in this setting;
7 To determine the molecular mechanisms leading to AMR, virulence factors and population diversity of *E. coli*.

## Methods

### Study setting

The study is conducted in primary healthcare clinics (PHCs) in Harare, Zimbabwe. PHCs provide acute primary care, including treatment for common infections. In addition, all PHCs have 1) maternity services to record and follow pregnancies in their catchment area and perform uncomplicated deliveries, 2) family planning and well-child services for growth monitoring and vaccinations, and 3) HIV services for regular follow-ups and provision of antiretroviral therapy.

The study sites are selected based on the number of clinic presentations, their catchment population and their location within Harare. The catchment population of the clinics, of over 800,000 people ^72^, belong mostly to the low-income strata and live in densely populated communities. The clinics selected serve the populations of the following suburbs: Budiriro, Glen View, Glen Norah, Mufakose, Highfields, Kuwadzana, Warren Park, Dzivarasekwa, Kambuzuma and Mbare.

The PHCs are primarily nurse-led and prescriptions are issued in accordance with national guidelines ^54^. UTIs are usually diagnosed clinically and first-line treatment is with a fluoroquinolone or amoxicillin. Patients purchase antimicrobials according to prescription either at the PHC pharmacy or at other pharmacies in the community. Study-specific procedures are performed by the study staff who are trained on the protocol and relevant study procedures.

### Study design

**Figure 2.**
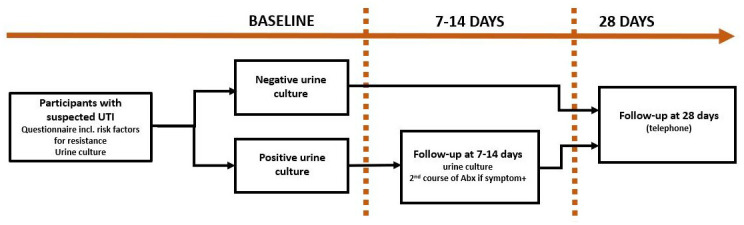
Outline of procedures at enrolment and follow-up. *UTI: urinary tract infection, Abx: antibiotics*.

This is a prospective cohort study enrolling adults (aged ≥18 years) who present with symptoms of UTI at PHCs in southwest Harare, Zimbabwe. Recruitment into the study will be over an 18-month period. All participants are followed up by telephone at 28 days post-enrolment. In addition, participants with a positive urine culture at enrolment are followed up between 7 and 14 days post-enrolment to provide a urine sample to assess for clearance of infection ( Figure 2).

**Figure 3.**
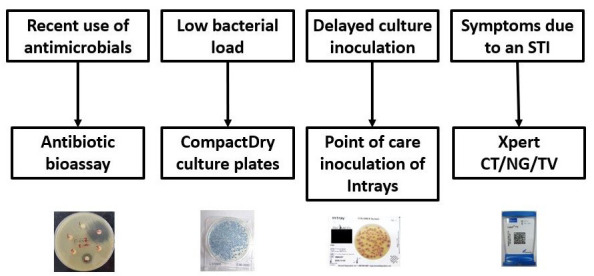
Evaluation of causes for negative urine cultures. *STI: sexually transmitted infections; CT: Chlamydia trachomatis; NG: Neisseria gonorrhoeae; TV: Trichomonas vaginalis*.

### Participant recruitment

Study staff screen and enrol participants according to the eligibility criteria. Recruitment is conducted five days per week during regular PHC opening hours. A total of 1500 participants with suspected UTIs will be enrolled. The reason for exclusion of screened participants is recorded.

### Eligibility

Patients are enrolled into the study if they fulfil all the inclusion criteria and do not have any of the exclusion criteria.

*Inclusion criteria:*

- age ≥18 years
- presenting with symptoms of UTI (≥2 of the following: dysuria, urgency, frequency, suprapubic pain and/or flank pain). The presence of at least two symptoms is required in order to exclude those who are more likely to have other conditions (e.g. sexually transmitted infections)
- onset of symptoms within two weeks prior to presentation
- presence of symptoms within the last 24 hours
- provision of written informed consent

*Exclusion criteria:*

- discharge from hospital within the previous 72 hours
- having a urinary catheter in-situ

Individuals with catheters are excluded because they are likely to represent a different population with more healthcare exposure, and are more likely to have previously been prescribed antimicrobials and to have infections with resistant organisms. These infections are more likely to be healthcare associated infections rather than community acquired infections, which is the focus of this study. Recruiting individuals with urinary catheters would therefore likely lead to an over-estimation of community-level resistance.

### Procedures at enrolment

***Clinical and demographic data collection.*** Data on age, sex, socio-economic status (measured using standardised asset ownership tool, education and employment of the head of the household ^73^), clinical history, prior health care seeking (traditional healer, private practitioner, pharmacy), and risk factors for AMR (prior antimicrobial use or hospitalization during the previous six months, comorbidities including HIV status, antiretroviral treatment, co-trimoxazole prophylaxis, chronic kidney disease and diabetes, current or recent pregnancy, recurrent UTIs) are collected using an interviewer-administered questionnaire, confirmed by patient-held records. Drug treatment (if any) and duration of treatment prescribed by the health care worker is recorded. Results of HIV tests, which are routinely carried out at the PHCs, are documented.

### Sample collection and laboratory processing

A midstream urine sample for microscopy, culture and antimicrobial susceptibility testing (AST) is collected in a sterile container. The samples are transported to the laboratory as soon as possible and if a prolonged time to delivery is anticipated (>4 hours), the samples are cooled to prevent overgrowth of contaminants.

Urine samples undergo dipstick and microscopy for leucocytes, and culture. A standardised sample volume (1 µl) is inoculated on chromogenic agar (Brilliance UTI agar, Oxoid, UK). Presumptive bacterial identification is performed according to the manufacturers’ instructions. A urine culture is considered positive if ≥10 ^3^ colony forming units (CFU)/mL are present with either pure culture or predominance of one organism ^74^. If cultures grow a non-uropathogen or if ≥2 organisms are isolated in the absence of a clear predominance of one organism, the culture is considered contaminated. When GNBs cannot be identified by colony appearance on chromogenic agar, biochemical testing with APIs (Analytical Profile Index, bioMérieux, France) is used. AST is performed using the Kirby-Bauer disc diffusion method and interpreted using EUCAST standards ^75^. Screening for ESBL production is performed according to EUCAST recommendations ^76^. Briefly, if resistance to cefpodoxime alone or ceftriaxone and ceftazidime is detected, double-disc synergy testing between a cephalosporin and clavulanic acid is performed. Similarly, for AmpC detection in isolates with cefoxitin and ceftazidime resistance, synergy testing between cefoxitin and cloxacillin is carried out. In addition, for isolates resistant to third-generation cephalosporins, the minimum inhibitory concentration for ceftriaxone is determined using E-tests (bioMérieux, France).

All bacterial isolates are stored on storage beads at -80°C. Stored *E. coli* isolates will be used to re-establish cultures on agar plates from which DNA will be extracted using the DNA QIAmp Mini Kit (Qiagen, Hilden, Germany). *E. coli* isolates will undergo whole genome sequencing to ascertain molecular determinants of AMR, virulence factors and population diversity. For whole genome sequencing, DNA libraries will be prepared using the Nextera XT DNA Sample Preparation Kit (Illumina, San Diego, USA) as per the manufacturer’s instructions. The libraries will be sequenced using the Illumina HiSeq platform (Illumina, San Diego, USA). Trimmed reads will be assembled into contigs using SPAdes and using a publicly available *E. coli* reference genome. Antimicrobial genotype and virulence gene prediction will be performed using ABRicate. Phylogeny will be determined using FastTree and viewed in FigTree.

### Evaluation of negative urine cultures

Pilot data and data from other studies from sub-Saharan Africa ^77, 78^ have shown that a large proportion of urine cultures from patients with symptoms suggestive of UTI are negative (60–75%) as compared to 25% in Europe ^79^. This may be due to various causes such as antimicrobial use prior to sample collection, low bacterial load, delayed sample inoculation leading to overgrowth of contaminants or depletion of pathogen, or symptoms due to sexually transmitted infections rather than UTIs. These alternative causes will also be investigated in a subset of participants from this study ( Figure 3).

To determine recent antimicrobial use, information on antimicrobials prior to clinic presentation and on co-trimoxazole use for HIV-positive individuals will be collected. In addition, urine samples will be evaluated for antimicrobial residues using a disc-diffusion adapted from Driscoll *et al.* ^80^. Low bacterial loads will be investigated using a highly sensitive culture system that is employed for testing coliform contamination of water and food (CompactDry EC, Nissui, Japan). Point of care inoculation of urine samples using InTrays (BioMed Diagnostics) will be used to determine if sample transportation delays may contribute to contamination and pathogen loss. The prevalence of sexually transmitted infections in Zimbabwe can be as high as 15–20% (unpublished data from Ferrand R.A. *et al*.). A subset of urine samples will be tested for gonorrhoea and chlamydia using Xpert CT/NG (Cepheid, Sunnyvale, CA, USA) and for trichomonas vaginalis using Xpert TV (for women only).

### Provision of routine care for study participants

Clinical care for study participants remains the responsibility of routine health care providers. Urine dipstick, microscopy and culture results are provided to the clinic health care workers, with advice from the study physician on management for complicated cases (such as prior treatment failure, isolation of multidrug resistant bacteria, pregnancy, or severe kidney or liver disease requiring dose-adjustment).

### Procedures at follow-up

Participants who have a positive urine culture at enrolment have a follow-up visit between 7 and 14 days after enrolment. Participants are asked to provide information on their symptoms, antimicrobial use and healthcare seeking since their enrolment. If a participant has not taken a prescribed antimicrobial, the reasons are also recorded. A second urine culture is collected to assess for clearance. If UTI symptoms have not resolved, a second course of antimicrobials based on results of the AST is considered, as clinically appropriate.

All participants, irrespective of enrolment culture results, are followed up by telephone 28 days after enrolment to assess clinical outcomes (i.e. symptom resolution, hospital admission, UTI symptom recurrence).

Participants due to come to the clinic for a follow-up visit will be notified in advance of their appointment. If participants are unable to come to the clinic, a home visit will be performed. For the telephone follow-up visit at 28 days, participants will be called on at least three separate occasions on two different days. If they cannot be reached by telephone, a home visit will be attempted. Loss to follow-up will only impact on the outcome analysis and was accounted for in the sample size calculation.

### Outcome measures

A UTI is classified as confirmed if the urine culture is positive with a recognised urinary pathogen or possible if the culture is negative or shows contamination. Bacteriological cure is defined as a negative urine culture following an initial positive urine culture. Clinical cure represents resolution of symptoms at the 7- to 14-day follow-up. Relapse is defined as the absence of a positive culture and symptoms after seven days but reappearance or re-presentation with symptoms within 28 days of the initial presentation. AMR to specific drugs and ESBL are defined using the EUCAST standards and guidelines for detection of resistance mechanisms ^75, 81^. Multidrug resistance is defined as resistance to one agent from at least three different antimicrobial classes ^82^.

### Data management

All processes related to data collection, management and storage are governed by standard operating procedures (SOPs) and follow the principles of Good Clinical Practice.

All participants are identified throughout the study by a unique identifying number that is assigned at recruitment using uniquely numbered and barcoded consent forms. Apart from age and sex no personal data are collected on the clinical report forms.

All data are collected and entered on handheld tablets into pre-designed forms using the Open Data Kit (ODK, www.opendatakit.org) software. Electronic data entry quality is ensured by real-time error capture, internal validation, consistency checks and stringent formatting constraints. For the instances when the data cannot be entered directly into the electronic form (e.g. laboratory results that are only available after 24–49h), data are recorded onto paper forms. Upon completion of the laboratory tests, the data from the paper forms are entered electronically. Paper forms are available in case of failure of electronic data entry in the field.

### Data analysis

Categorical variables will be analysed using counts and percentages and continuous variables using means/medians and standard deviations/interquartile ranges. The proportions of study participants with a positive, contaminated and negative urine culture will be determined. Prevalence and 95% confidence intervals will be presented for each causative organism and for resistance to antimicrobials. Univariate associations between risk factors and the presence of first-line and first- and second-line resistance and clinical and bacteriological outcome will be assessed using the χ ^2^ test for categorical variables. STATA (version 14, Stata-Corp, TX, USA) will be used for data analysis.

For the primary objective (association between HIV infection and ESBL presence in *E. coli*), a logistic regression model will be built, which will include age and sex as the pre-specified confounders and which will be controlled for the other variables which show an association in the univariate analysis (e.g. recent hospitalization, recent antimicrobial use, pregnancy). The molecular mechanisms of resistance and virulence factors will be reported in a descriptive analysis.

### Sample size estimates

The sample size calculations used the following assumptions from published studies and a pilot study: 30% of urine cultures are positive ^40, 41^, 90% of the positive cultures yield *E. coli*, ESBL prevalence in *E. coli* is 15% in HIV-negative and 30% in HIV-positive individuals, 25% of participants are HIV-positive and 90% of study participants know their HIV status. In order to determine if there is a difference in proportions of ESBL-producing *E. coli* between HIV-positive and HIV-negative individuals, 1404 participants presenting with symptoms of UTI would need to be recruited into the study, of which 405 would be included in the primary outcome analysis.

For the clinical outcome analysis, UTI with a bacterial strain showing AMR (defined as resistance to ciprofloxacin or amoxicillin according to Zimbabwean guidelines) ^71^ will be considered as the exposure, and complete resolution of symptoms (clinical cure) at the day 7 follow-up visit the outcome. Preliminary data from this study have shown that the prevalence of AMR in UTI isolates is 83%. The assumptions used will be: 500 cultures are positive, loss to follow up is 10%, 80% of isolates are resistant to amoxicillin or ciprofloxacin, 30% of participants with symptoms have a positive culture, and 20% of patients did not take antimicrobials. Estimating a positive impact of treatment on clinical cure in participants without AMR of 80–90% and 40–50% in participants with AMR, the study will have >80% power to detect a difference between the groups.

### Study status

The study began recruiting participants in June 2019 and recruitment is ongoing.

## Discussion

Although there are indications that PLWH are at increased risk for infections with resistant organisms as compared to the general population, most studies have focused on gram-positive pathogens such as *Streptococcus pneumoniae* ^83^ and *Staphylococcus aureus* ^84^. The ARGUS study will investigate if there is an association between HIV infection and AMR in GNB causing UTIs in Harare. The study will also investigate other risk factors for AMR in this setting. Results may contribute to the development of specific treatment recommendations based on the risk of AMR. Furthermore, the study will provide important data on the prevalence of AMR in community-acquired infections caused by GNB in this setting which will inform antibiotic prescribing guidelines, as well as the development of strategies to prevent further dissemination of resistance. The findings of this study are especially important since data on priority organisms for AMR surveillance from a large number of clinics across Harare will be collected. The information on outcomes of infections will guide the design of future management algorithms including identification of patients at risk for persistent infections and for complications.

The study is limited by its recruitment from a single city in Zimbabwe and therefore results might not be generalizable to the whole country which has a predominantly rural population. However, participants are recruited from ten PHCs across Harare and are therefore representative of the urban population. Individuals accessing healthcare at the clinics are required to pay a consultation fee. Due to the economic challenges in Zimbabwe, there has been an increase in consultation fees alongside rapid inflation. Therefore, individuals with mild symptoms may not access the clinic and will therefore not be included.

### Ethics and dissemination

The study was approved by the ethics committees of the Medical Research Council of Zimbabwe (MRCZ/A/2406), the London School of Hygiene and Tropical Medicine (Ref. 16424), and the Biomedical Research and Training Institute. The study was granted permission from the City of Harare Department of Health. All study participants have provided/must provide written informed consent prior to enrolment into the study.

The study results will be disseminated to healthcare workers at the clinics through leaflets and dissemination meetings with the aim to enhance understanding, discuss the findings and ultimately improve future patient management. Significant microbiological results from individual patients will be reported to the attending healthcare worker as soon as they are available, in order to optimise treatment for individual patients. A report of the study results will be provided to the PHC healthcare workers, the Ministry of Health and Child Care, Harare City Health and other relevant stakeholders and policy makers. Data from this study may be used to inform treatment guidelines in order to improve patient management. The results of this study will be presented at national and international conferences to a wider audience and will be published in peer-reviewed journals.

## Data availability

No data are associated with this article.
