# Supplementary material for: Antimicrobial Resistance in Gram-negative bacteria from Urinary Specimens: a study of prevalence, risk factors and molecular mechanisms of resistance (ARGUS) in Zimbabwe – a study protocol
Source: Wellcome Open Res. 2020 Jun 12;5:140. [Version 1] doi: 10.12688/wellcomeopenres.15977.1 (PMC7551514; doi:10.12688/wellcomeopenres.15977.1)
Supplement: Peer review history

# Peer review: Reviewer response for version 1

**DOI:** 10.21956/wellcomeopenres.17522.r40340

**Published:** 2020-10-12

**Authors:** Nabil Karah

**Affiliations:** 1 Department of Molecular Biology, and Umea Centre for Microbial Research, Umea University, Umeå, Sweden

The manuscript describes a study protocol on the prevalence, risk factors, and molecular mechanisms of AMR in Zimbabwe. Both the study design and the manuscript were very well done.

**Comment 1**

- What equation have the authors used to calculate the sample size? The equation reference should be cited.

 

**Comment 2**

- The high rate of negative cases (60–75%) could also be due to inappropriate ordering and acquisition of urine cultures (Redwood *et al*., 2018) ^1^. The fact that this service is free-of-charge could be an appealing factor.

 

**Minor comments:**

- “to treat infections successfully.” instead of “to successfully treat infections.”  
- “Gram-negative” instead of “gram-negative” (correct throughout the manuscript).
- to investigate the prevalence and determine the molecular mechanisms of AMR in.
- “and its impact on clinical outcomes” instead of “how AMR impacts on clinical outcomes”.
- “to guide treatment recommendations” instead of “to inform policy and development of treatment recommendations”.
- Maybe “understanding of the molecular epidemiology of successful pathogens and the prevalence of clinically significant AMR genes in Zimbabwe”.
- has, however, been accompanied.
- Delete “At an individual level”.
- “The risk factors ...” instead of “Other risk factors …” 
- Maybe re-write as: Underlying co-morbid conditions and … are major risk factors for infections due to resistant organisms.
- What do you mean by “healthcare contact”? Be more specific.
- However, few African countries have so far contributed data to these networks.
- health care systems in sub-Saharan Africa, infections are 
- “antimicrobials as ‘access’, ‘watch’ or ‘reserve’ and for” instead of “antimicrobials as ‘watch’, ‘access’ or ‘reserve’ and for“
- According to this classification, …
- “access to amikacin or carbapenems” instead of “access to amikacin or to carbapenems”
- severely affected, with most countries 
- Re-write a correctly structured sentence instead of “However, co-trimoxazole has been reported to increase carriage of resistant organisms in PLWH”
- Delete “which are transferrable between bacterial species.”
- Delete “i) to determine if infections in PLWH are more commonly due to antimicrobial resistant organisms, compared with infections in individuals without HIV infection;”
- Re-write a clear sentence instead of “ii) to explore the prevalence of and underlying molecular mechanisms for AMR in GNB causing UTIs,”
- Change the order of sentences as following: Primary healthcare clinics (PHCs) in Zimbabwe provide acute primary care, including treatment for common infections. In addition, all PHCs have 1) maternity services to record and follow pregnancies in their catchment area and perform uncomplicated deliveries, 2) family planning and well-child services for growth monitoring and vaccinations, and 3) HIV services for regular follow-ups and provision of antiretroviral therapy. The study is conducted in “add the exact number” selected PHCs in Harare, Zimbabwe. The study sites are selected based on the number of clinic presentations, their catchment population and their location within Harare. The catchment population of the clinics, of over 800,000 people ^72^, belong mostly to the low-income strata and live in densely populated communities. The selected “PHCs” serve the populations of the following suburbs: Budiriro, Glen View, Glen Norah, Mufakose, Highfields, Kuwadzana, Warren Park, Dzivarasekwa, Kambuzuma and Mbare.
- Delete “for microscopy, culture and antimicrobial susceptibility testing (AST)”
- A UTI is classified as “confirmed” if the urine culture is positive with a recognised urinary pathogen, or “possible” if the culture is negative or shows contamination. 

Is the study design appropriate for the research question?

Yes

Is the rationale for, and objectives of, the study clearly described?

Yes

Are sufficient details of the methods provided to allow replication by others?

Yes

Are the datasets clearly presented in a useable and accessible format?

Not applicable

Reviewer Expertise:

Laboratory Diagnostics Specialist

I confirm that I have read this submission and believe that I have an appropriate level of expertise to confirm that it is of an acceptable scientific standard.

## References

[1] : Reducing unnecessary culturing: a systems approach to evaluating urine culture ordering and collection practices among nurses in two acute care settings. *Antimicrob Resist Infect Control*.2018;7: 10.1186/s13756-017-0278-9 4 10.1186/s13756-017-0278-9 29340148PMC5759376

# Peer review: Reviewer response for version 1

**DOI:** 10.21956/wellcomeopenres.17522.r39172

**Published:** 2020-07-20

**Authors:** Susanna Dunachie

**Affiliations:** 1 Centre for Tropical Medicine and Global Health, University of Oxford, Oxford, UK; 2 Mahidol-Oxford Tropical Medicine Research Unit, Bangkok, Thailand

This article describes the study protocol for a prospective study of prevalence, risk factors, and mechanisms of resistance of AMR in Gram-negative bacteria in urine from primary care clinics in Harare, Zimbabwe.

 The article is well written with a very nice review of the (limited) literature on AMR in sub-Saharan Africa and the excellent Figure 1. 

 The proposed study is sound and will be a very welcome addition to our understanding of AMR in Zimbabwe. Given the paucity of high-quality microbiological data that is representative of the wider community, this data will also have relevance to other low-income countries with similar population structures and health care provision. The opportunity to explore the high rates of culture-negative urines in this setting by measuring urinary antibiotics, maximising sensitivity, trialling point of care tests, and testing for STIs with cutting edge diagnostics is especially welcome.

 Some minor points: 

1. There is still some debate about the evidence base on what drives AMR - advise softening the 1st sentence of the 2nd paragraph in the Introduction or at least citing some eminent opinion. 
2. Introduction 4th paragraph: A major issue with existing microdata from LMIC settings is data quality. Advise mentioning this, and a sentence in your discussion on how the micro lab to be used demonstrates quality (use of EUCAST already described, can add the use of standard controls, plus the fact that the isolates will be sequenced. See ref (1)). You can also add that existing AMR data is biased towards the data from the more wealthy population due to more microdata coming from private labs and cities. Your community-based design will capture a much more representative population than most of the published literature.
3. The data analysis plan is good. Consider including as a variable in the screening univariate analysis some metrics for each clinic such as clinic size and district as I imagine there will be heterogeneity in the types of clinics. Do all the clinics have the same funding source? How many clinics will be used?
4. Very minor, but most international style guides advise using a capital G from Gram, after the Danish microbiologist. However, I do concede that some US journals style guides prefer the lower case g. 

Overall this is the sort of well thought-out, comprehensive study in a representative population that is very much needed for understanding the global burden and mechanisms of AMR.

Is the study design appropriate for the research question?

Yes

Is the rationale for, and objectives of, the study clearly described?

Yes

Are sufficient details of the methods provided to allow replication by others?

Yes

Are the datasets clearly presented in a useable and accessible format?

Yes

Reviewer Expertise:

Global health researcher and clinical microbiologist with experience of working in low and middle income settings. Highly experienced in reviewing data on AMR from LMIC settings

I confirm that I have read this submission and believe that I have an appropriate level of expertise to confirm that it is of an acceptable scientific standard.

## References

[1] : Microbiology Investigation Criteria for Reporting Objectively (MICRO): a framework for the reporting and interpretation of clinical microbiology data. *BMC Medicine*.2019;17(1) : 10.1186/s12916-019-1301-1 10.1186/s12916-019-1301-1 30922309PMC6440102
